# Impact of Digital Device Use on Neck and Low Back Pain Intensity among Nursing Students at a Saudi Government University: A Cross-Sectional Study

**DOI:** 10.3390/healthcare10122424

**Published:** 2022-11-30

**Authors:** Nermen A. Mahmoud, Ahmad H. Abu Raddaha, Donia E. Zaghamir

**Affiliations:** 1 Department of Nursing, College of Applied Medical Sciences, Prince Sattam Bin Abdulaziz University, AlKharj 11942, Saudi Arabia; 2 Department of Medical Surgical Nursing, Faculty of Nursing, Kafrelsheikh University, Kafr El Sheikh 6860404, Egypt

**Keywords:** digital devices, disability, neck pain, nurses, students

## Abstract

As digital technology and online activities have become more widely accessible over the past few years, information and communication technology have grown in importance in all students’ lives. Most of them routinely use digital devices for a range of activities, primarily for online learning, activities, assignments, conversing, and Internet browsing. This study elucidated the relationship between neck and low back pain intensity and the use of digital devices among Saudi nursing students. A cross-sectional descriptive exploratory research design was applied using a convenience sample of 120 nursing students enrolled in an undergraduate nursing program at a Saudi government university located in Riyadh, the capital city of Saudi Arabia. A valid and reliable self-administered survey was employed. Data about sociodemographic characteristics, the Neck Disability Index and the Roland–Morris Disability Questionnaire were collected. The participants reported 9.1 ± 4.6 study hours on average per week. A total of 82.5% of them do not receive enough exercise, and 87.5% indicated daily use of digital devices. Around half (54.2%) of participants reported having neck pain of a mild intensity. About 60% of the participants regularly shift positions to rest their backs, 39.2% are only able to stand for limited periods due to back issues, and 39.2% expressed concern for others about what might happen to their health. Although there was an association between neck pain intensity and the age or gender of all research participants, there was a significant association between neck pain severity and marital status (χ^2^ = 15.226, *p* = 0.019). Our findings suggest that nursing students should maintain neutral neck and back postures on a regular basis to reduce pain, which could be attributed to extensive reading on digital devices.

## 1. Introduction

Information and communication technology have become increasingly significant in all students’ lives as the availability of digital technology and online experiences has increased over the past several years. To combat the pandemic caused by coronavirus disease 2019 (COVID-19), the teaching community has benefited from the use of small computer devices and technology [1]. Electronic device usage has become widespread among college students. The majority of them frequently utilize digital devices for a variety of purposes, primarily for online learning activities, assignments, chatting, and Internet surfing [2].

There is no doubt that utilizing digital devices has many advantages, but if they are utilized inappropriately, they can also pose serious health risks that include musculoskeletal and ocular issues. Musculoskeletal pain is a serious health issue that affects both adults and children [3]. When using a laptop, many college students noted experiencing musculoskeletal pain, with neck and shoulder pain being more common [4]. Further complaints included backache and tingling in the fingers. In a recent study among students in a college of medicine, Nagwa et al. [5] found that the usage of desktop computers has led to complaints of neck and shoulder pain and numbness or tingling in the fingers, and caused loss of productivity, sick leave, and even disability. Furthermore, most people eventually experience back, neck, and shoulder pain, but few do so for extended periods. The average lifespan is increasing, and low back discomfort has long been a key contributor to disability; almost everyone experiences low back pain at some point in his or her lifetime, with a lifetime prevalence of 70–80% [6].

Because people are more likely to look down and slouch when using touch-screen digital devices (such as smartphones and tablets), neck and backaches may arise more frequently when using them [7,8]. Specifically, back pain and neck pain result from neck flexion, which occurs when a person bends their neck in a downward position for a prolonged period while looking at smartphones or other digital devices without proper training or in uncomfortable postures, and is affected by the duration and frequency of these devices usage [9].

Several smartphones and other hand-held devices require the user to maintain a low head posture for an extended period while holding these devices in front of them. This causes the user’s head to lean forward, flattening the cervical lordotic curve. Neck and back pain syndromes may develop because of forward head posture [6]. Moreover, using a smartphone while standing still and holding it with an unsupported arm may cause an aberrant alignment of the neck and shoulders. Users of smartphones must bend their necks to view the small screens because they often are held close to the lap, which increases activity in the neck muscles and overloads the neck and shoulders, causing pain and tiredness [10,11]. In Saudi Arabia, a previous study among 516 university students showed that 98% of people used electronic devices, 92% of whom used smartphones and iPads for amusement, 32% of whom used them for studying, and 59.1% reported experiencing neck or shoulder pain while using electronics [12]. An Ethiopian study among medical students reported that 46–52% of participants experienced shoulder issues, while 68% reported neck symptoms due to mobile devices usage [4].

Given the scarcity of published research in Saudi Arabia on the effect of using digital devices on the severity of neck and low back pain, the purpose of this study was to assess how the use of digital devices affects the intensity of cervical and low back pain among undergraduate nursing students. The study addressed the following objectives:

1. Describe how nursing students interact with their electronic devices.
2. Determine the overall neck disability level of the nursing students.
3. Define any functional limitations in nursing students associated with low back pain.
4. Examine relationships between nursing students’ sociodemographic characteristics and their neck disability level scores.

## 2. Methods

### 2.1. Design

A cross-sectional descriptive exploratory research design was used.

### 2.2. Setting and Sampling

Undergraduate nursing students who met the inclusion criteria were invited to participate in the study at a university located in Riyadh, the capital city of Saudi Arabia. A convenience sampling technique was employed.

Inclusion criteria: Being a male or female undergraduate nursing student at the time of data collection and being able to read English were the inclusion criteria.

Exclusion criteria: Students who had a known medical condition (recent trauma or surgery) around the neck and any spinal vertebral deformity were not allowed to take part in this study.

### 2.3. Sample Size

The sample size was estimated using the formula developed by Charan and Biswas [13], as shown below:

$$n=\frac{{\left(Z_{1}-\frac{\alpha }{2}\right)}^{2}\cdot {\mathrm{SD}}^{2}}{d^{2}}$$

Given a level of significance of 5% and power of study of 80%, we can rely on the literature [14] using Z_1_ − α/2 (the standard normal variate) of 1.96, the SD (standard deviation) of the variable, and d for absolute error or precision. The sample size needed for the study was 120, according to the formula.

$$n=\frac{{\left(1.96\right)}^{2}\cdot {\left(7.0\right)}^{2}}{{\left(1.253\right)}^{2}}=119.9$$

### 2.4. Instruments

A self-administered survey was made available online using Google Form. Following the completion of an Informed Consent Form (ICF) by the participants, the survey contained elements to gather sociodemographic characteristics about age, sex, year of study, exercise, duration of computer usage per day (hours), usual position of study, and type of chair used. The researchers developed this section after evaluating the study of Cankurtaran and Beverland [15], who highlighted the importance of assessing key characteristics to aid in understanding the situation and developing solutions while managing risks, particularly those that may have an impact on health. Three experts in the field reviewed the section’s content validity, clarity, and appropriateness.

Vernon Mior’s [16] Neck Disability Index (NDI) was used. It is a widely known tool for measuring neck pain, and has achieved a high degree of reliability and face and concurrent validities consistency. It has a 10-item Likert scale with a range of 0 (No activity limitation) to 5 (Worst activity limitation). Points summed to a total score. The total score is between 10 and 50, with a high score indicating greater neck disability [16]. In this study, the Cronbach’s alpha was 0.90, indicating high reliability. The 22-item Roland–Morris Disability Questionnaire was adapted from Longo et al. [17] to evaluate functional limitations due to low back pain. It has high internal reliability, content and construct validities [13]. In this study, the Cronbach’s alpha was 0.89, indicating high reliability.

### 2.5. Ethical Considerations

The participants were invited to participate in the study on a voluntary basis after the researchers obtained ethical approval from a Research Ethics Committee at a governmental university. The participants were provided with written informed consent outlining the study’s objectives.

### 2.6. Procedure

The researchers started data collection by sending a Google Form to students via WhatsApp invitations and asking them to respond. The timeframe of data collection was from April 2022 to July 2022. The student provided his or her agreement to participate in the study during this time.

### 2.7. Statistical Analysis

Upon completion of data collection, data were entered into the analysis software to be analyzed. The IBM Statistical Package for Social Sciences Statistics (IBM SPSS Statistics), version 29, was used for data analysis. For all statistical tests conducted, a level of significance of 0.05 was chosen.

The continuous data had a normally distributed distribution and were expressed as mean ± standard deviation (SD). Categorical data were expressed using numbers and percentages. The Chi-square test was used to compare variables with categorical data. The internal consistency (reliability) test was computed for the questionnaires used in the study. There were no missing data found.

## 3. Results

Given that the study employed convenience sampling, a total of 120 nursing students voluntarily completed the questionnaire, matching the calculated needed sample size. At the time of data collection, there were 486 students enrolled in the participating undergraduate nursing program.

Table 1 shows that 55% of the study participants were under the age of 22, with a mean age of 21.4 (±1.7 SD) years. A total of 88% of the participants were female, and 89.2% were single. Furthermore, 25.8% of students had entered the eighth academic level.

Most (92.5%) of the participants use the right hand as their dominant side. Additionally, with a mean of 9.1 (±4.6 SD) study hours per week, 82.5% of the participants reported that they do not exercise regularly and 50% had a normal body mass index (Table 2).

Table 3 depicts that 87.5% of participants use digital devices every day. All of them use smartphones, and 89.2% use tablets. Furthermore, 54.2% of them used digital devices when they were younger than thirteen years old, 70.0% expressed that they use digital devices while lying in bed, 60% of participants spend less than six hours per day using computers, 50.8% spend less than ten hours per day using phones, and 51.7% spend less than three hours using other digital devices.

**Figure 1:**
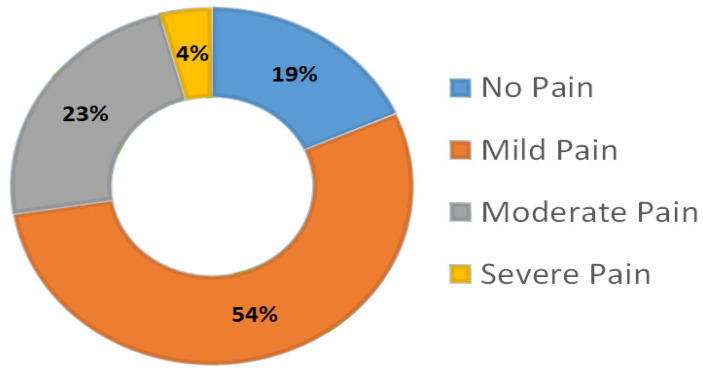
Distribution of Total Neck Disability Index score of the students.

Figure 1 shows that 54% of the participants suffered from a mild intensity level of neck pain. Around 60% of participants regularly change positions to rest their backs, 39.2% can only stand for brief amounts of time due to a back problem, 39.2% express their worry for others regarding their health, and 31.7% rub or pick at painful or uncomfortable areas of their body. On the other hand, only 6.7% expressed that they dress slower than usual because of their back problem (Table 4).

Table 5 shows that there is no significant correlation between neck pain and age or gender among the study participants, but there is a significant association between neck pain intensity and marital status.

## 4. Discussion

Regarding the digital devices types that were used and their usage hours, the study showed that almost all of the students use all the electronic devices (smartphones, iPads, and computers) daily for around six to ten hours. This finding was consistent with a prior study from Bicen and Kocakoyun [18], who found that most students utilize mobile device applications on a regular basis. The increased number of hours spent using digital devices raises the risk of health hazards.

The different levels of education rely on online learning, especially after the COVID-19 pandemic, by using computers, smartphones, tablets, iPads, etc. The use of these digital devices is a major issue to students and may lead to physical discomfort, especially neck and back pain. In 2022, Cheung, Lai and Yip’s study [19] found that 46.3% of students used computers for less than one hour per day. Most of our study participants appear to use mobile digital devices on a daily basis, increasing their risk of neck and back pain.

Half of the current study’s nursing students had normal body mass indexes. A previous study by El-Bidawy et al. [20] on the medical college students at a Saudi university showed a positive correlation between being overweight and experiencing low back pain. They reported that the average body mass index of the medical student participants was 26.39 (±6.16).

Concerning their neck condition, our study participants had just mild pain when performing personal care and during reading. The findings of this study are consistent with the study findings of Hasan et al. [21]. These neck pain complaints and their impact on daily life reduce students’ productivity and raise the number of sick days they take [5].

Neck pain has a great impact on the activities of daily living and may affect the individuals’ participation and lead to work disabilities. About half of the nursing students experienced minor discomfort when reading, and some had slight trouble focusing because of prolonged neck pain. These are in line with the findings of Tanveer [22] and Kim [23], who reported that the respondent had neck pain.

The current study also revealed no correlation between neck pain and the age or gender of the participants. This outcome is consistent with Kim’s [23] finding that no significant differences in their ages and demographics were observed. However, we found a significant correlation between neck pain intensity and marital status, where single participants suffered less than married and divorced ones.

With regard to low back pain, which is one of the leading causes of disability worldwide [23], the current study revealed that around 60% of the participants were changing their positions frequently to try to rest their back. From the researchers’ view, when people are in pain, they try to change their position in order to relax their muscles and feel more at ease.

Around 40% of the participants were unable to stand for an extended period due to back pain. This finding is in line with Eloi et al. [24] who reported that 75.8% of students from all levels of a medicine program experience chronic pain that affects their positions and abilities to stand for an extended period of time. In a similar vein, El-Bidawy et al. [20] reported that 60% of the sample reported that rest helped with pain relief.

The age range of study participants and the number of female students were comparable to past studies [12,25]. However, we found no significant relationships between neck pain and either age or gender among the study participants. The young age of the participants in both studies may influence how this is perceived. It is recommended, however, that participants and the public receive education about proper digital device use through the media and educational institutions in order to reduce the risk of back pain while using smartphones and other hand-held digital devices. On the other hand, the presence of other confounding variables, such as family roles among married participants and their effect on back pain, may influence the significance of the relationship between back pain and marital status.

### Strengths and Limitations

The study has the following strengths: (1) the large sample came from Riyadh, Saudi Arabia’s capital and largest governorate, and (2) the nursing students came from a variety of geographic regions in Saudi Arabia. Some limitations in this study restrict the generalizability our findings. These are the limitations: (1) the cross-sectional design of the study can only provide a snapshot of the situation in our sample as it is not prospective in nature, and (2) the use of self-administered questionnaires can lead to social desirability bias.

## 5. Conclusions

There have been a limited number of studies that addressed digital device use among nursing students. More than two-thirds of participants use digital devices on a daily basis, including while lying in bed. As highlighted, almost half of the participants reported having neck pain of a mild intensity, and 82% of them reported that they do not exercise regularly. Furthermore, more than one-third of the sample reported that they stand for short periods due to back problems.

A significant association between neck pain severity and marital status was elucidated. Single participants suffered less than married and divorced ones. When it comes to spreading awareness about the health and safety dangers linked with computer use, nursing students should receive top priority. Our findings can be used to establish policies and interventions that attempt to lower the risk and onset of low back pain. We recommend conducting longer-term studies in various age- and sex-specific populations.

## Figures and Tables

**Table 1:** Number and distribution of the sociodemographic characteristics of the students.

Demographics	*n*	%	Mean	SD
Age (Years)			21.4	1.7
<22	66	55.0		
22 or More	54	45.0		
Gender				
Male	32	26.7		
Female	88	73.3		
Marital Status				
Single	107	89.2		
Married	9	7.5		
Divorced	4	3.3		
Number of Children				
None	116	96.7		
1	2	1.7		
2 or More	2	1.7		
Academic Level				
Third	10	8.3		
Fourth	26	21.7		
Fifth	16	13.3		
Sixth	17	14.2		
Seventh	20	16.7		
Eighth	31	25.8		

**Table 2:** Number and distribution of the life style of the students.

Items	*n*	%	Mean	SD
Dominant side			9.1	4.6
Right	111	92.5		
Left	9	7.5		
Do you smoke?				
Yes	19	15.8		
No	101	84.2		
Do you exercise regularly?				
Yes	21	17.5		
No	99	82.5		
Body mass index (kg/m^2^)				
Underweight	24	20.0		
Normal	60	50.0		
Overweight	25	20.8		
Obese	11	9.2		
Average number of hours of study per week				
<10	61	50.8		
10 or More	59	49.2		
Pain lasts for more than three months				
Yes	41	34.2		
No	79	65.8		

**Table 3:** Number and distribution of digital devices usage and spent hours.

Items	*n*	%
Do you use a digital device daily?		
Yes	105	87.5
No	15	12.5
Do you own a personal laptop or computer?		
Yes	92	76.7
No	28	23.3
Do you use a smartphone?		
Yes	120	100.0
No	0	0.0
Do you use a tablet?		
Yes	107	89.2
No	13	10.8
Age when you first time used digital device (Years)		
<13	65	54.2
13 or More	55	45.8
Position commonly used while using digital devices		
Sitting on Desk	5	4.2
Sitting on Chair	5	4.2
Floor	26	21.7
Lying in bed	84	70.0
Time spent on the computer (Hours/day)		
<6	72	60.0
6 or More	48	40.0
Time spent on the phone (Hours/day)		
<10	61	50.8
10 or More	59	49.2
Time spent on other digital devices (Hours/day)		
<3	62	51.7
3 or More	58	48.3

**Table 4:** Number and distribution student functional limitation due to low back pain.

Items	Yes	
	*n*	%
I stay at home most of the time because of my back pain	15	12.5
I walk more slowly than usual because of my back problem	21	17.5
I change position frequently to try and rest my back	73	60.8
Because of my back problem, I don’t do any of the chores I normally do at home	16	13.3
Because of my back problem, I use the handrail to go upstairs	13	10.8
Because of my back problem, I have to hold on to something to get out of a comfortable chair	16	13.3
I get dressed slower than usual because of my back problem	8	6.7
I only stand for short periods because of my back problem	47	39.2
Because of my back problem, I try not to bend or kneel	13	10.8
I find it difficult to get up from the chair because of my back problem	13	10.8
My back or legs hurt almost all the time	21	17.5
I find it hard to turn over in bed because of my back problem	17	14.2
I find it difficult to put my socks on because of the pain in my back	11	9.2
I only walk short distances because of my back problem or leg pain	17	14.2
I sleep less because of my back problem	14	11.7
I avoid strenuous work around the house because of my back problem	17	14.2
Because of my back problem, I’m more nervous and ill-tempered with people than usual	19	15.8
Because of my back problem, I am going upstairs much slower than usual	13	10.8
I stay in bed most of the time because of my back	19	15.8
I keep rubbing or picking at painful or uncomfortable areas of my body	38	31.7
Because of my back problem, I do less daily work at home than I usually do	22	18.3
I often express my concern to others about what might happen to my health	47	39.2

**Table 5:** Association between the sociodemographic characteristics and Neck Disability Index score.

	No Pain		Mild Pain		Moderate Pain		Severe Pain		Chi-Square	
	*n*	%	*n*	%	*n*	%	*n*	%	χ^2^	*p*
Age (Years)									7.640	0.054
<22	8	36.4	43	66.2	13	46.4	2	40.0		
22 or More	14	63.6	22	33.8	15	53.6	3	60.0		
Gender									4.280	0.233
Male	9	40.9	16	24.6	7	25.0	0	0.0		
Female	13	59.1	49	75.4	21	75.0	5	100.0		
Marital Status									15.226	0.019 *
Single	15	68.2	62	95.4	25	89.3	5	100.0		
Married	4	18.2	3	4.6	2	7.1	0	0.0		
Divorced	3	13.6	0	0.0	1	3.6	0	0.0		

* *p* < 0.05.

## Data Availability

The data presented in this study are available on request from the corresponding author. The data are not publicly available due to privacy restrictions.
